# Selenium in the Preterm Infant: Are We Supplementing Enough in This Vulnerable Population?

**DOI:** 10.3390/nu18081271

**Published:** 2026-04-17

**Authors:** Jeffrey V. Eckert, Lynette K. Rogers, Trent E. Tipple, Maxwell Mathias

**Affiliations:** Neonatal-Perinatal Medicine, Department of Pediatrics, University of Oklahoma Health, Oklahoma City, OK 73104, USA; jeffrey-eckert@ou.edu (J.V.E.); lynette-rogers@ou.edu (L.K.R.); trent-tipple@ou.edu (T.E.T.)

**Keywords:** prematurity, selenium, trace elements, oxidative stress

## Abstract

Selenium (Se) is an essential trace element. The bioactivity of Se arises from its incorporation into the 21st amino acid, selenocysteine (Sec). Twenty-five human genes have been identified that encode selenoproteins, each of which contains at least one Sec residue. Selenoprotein functions include antioxidant responses, thyroid hormone synthesis, and maintenance of cellular redox homeostasis. Due to its role in critical cellular functions, Se deficiency is associated with morbidities of the cardiovascular system and connective tissue in regions of countries with low soil Se content. While these morbidities are geography-specific and have been mitigated in adults through public health interventions, preterm infants remain susceptible to Se deficiency worldwide. Infants born preterm are deprived of fetal Se accrual in the 3rd trimester of pregnancy, a deficiency compounded by higher Se needs than term infants and older infants and dependence on parenteral nutrition (PN) and fortification. In addition, the composition of selenoproteins and selenometabolites in human milk is different from that in formula and PN, yet little is known about the biological impact of these differences. The knowledge gap in optimal Se supplementation is reflected in discrepant guidelines between North American and European/Chinese nutrition societies, whose recommended Se supplementation in preterm infants differs by more than 2-fold. In this review, we describe the biosynthesis, metabolism, and maternal-fetal transfer of Se. In addition, we address how developmentally regulated aspects of metabolism may impact how preterm infants respond to supplementation with different forms of Se. Lastly, we highlight current challenges and recommendations for optimizing Se levels in neonates based on available data.

## 1. Introduction: What Is the Biological Function of Selenium?

Most Se is present at birth and is accrued in the third trimester of pregnancy [1,2]. Thus, infants born preterm may be as much as 17 times as likely to be Se deficient when compared to term infants [3,4]. Preterm infants also frequently require parenteral nutrition, and nearly all require fortification of human milk or formula for adequate growth, both of which contain inorganic Se forms not present in human milk [5]. In order to critically appraise the current recommendations and published data on Se supplementation in preterm infants, we review the biology of Se and selenoproteins with a focus on unique aspects of preterm physiology.

**Figure 1:**
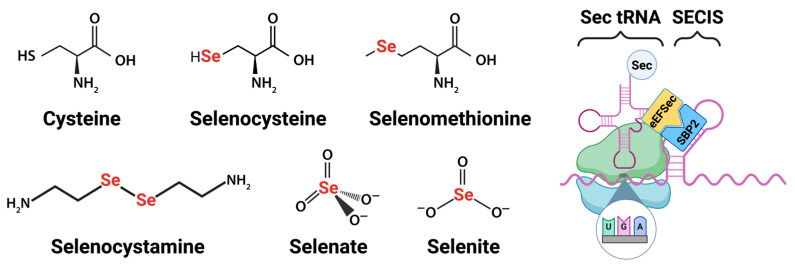
(**Left**) Cysteine structure and bioactive forms of selenium found in food, human milk, dietary supplements, and parenteral nutrition. (**Right**) Canonical selenopeptide synthesis requires 1. tRNA^sec^ binding a UGA codon, 2. proximity to a looped SECIS element, 3. eEFSec binding tRNA^sec^, and 4. SBP2 binds the looped SECIS element of selenoprotein mRNA. Created in BioRender. Mathias, M. (2026) https://BioRender.com/oaog0n6.

The bioactivity of Se is due to the high reactivity of selenium-containing proteins (selenoproteins), which contain the amino acid Sec, a modified cysteine with a side chain containing Se in the place of sulfur (Figure 1). The side chain pK_a_ of ~5.2, compared with that of cysteine (~8.3), makes Sec far more reactive than cysteine. At a physiologic pH of 7.4, >99% of Sec is in its selenolate (Se^−^) form [6,7,8]. These features are responsible for the reductive (electron-donating) potential of selenoproteins and account for their catalytic activity [6,7]. Sec synthesis and insertion into polypeptide sequences require multiple specialized components: a Sec-bound transfer RNA (tRNA^sec^) encoded by the *Trsp* gene, a tRNA^sec^-specific elongation factor (eEFSec), a looped Sec insertion sequence (SECIS) in selenoprotein mRNA, and a coordinating SECIS binding protein (SBP2) [9,10]. In contrast to classical polypeptide synthesis, Sec is first synthesized directly on its transfer RNA [10]. During selenoprotein synthesis, the UGA codon, usually a “stop” codon, in proximity to a SECIS element, binds tRNA^sec^ and adds Sec to the growing polypeptide of selenoproteins [11,12]. Selenoproteins are almost exclusively responsible for the biological effects of Se [13].

While there are 25 identified human genes encoding selenoproteins, many factors contribute to a far higher number of selenoproteins in the human body [14]. The low sequence conservation of SECIS elements, isoforms of SECIS-binding proteins, and selenoprotein mRNA splice variants leads to significant heterogeneity in the selenoproteome [15,16,17]. Advances in selenoprotein purification, mass spectrometry, and computational tools may identify additional selenoproteins and selenoprotein functions [18]. Known selenoprotein functions include maintenance of redox homeostasis, protein folding and endoplasmic reticulum (ER) function, Sec transport, and thyroid hormone synthesis (Table 1) [19,20]. Among the seleno-antioxidant enzymes, the most abundant is the glutathione peroxidase family (GPx), which converts hydrogen peroxide (H_2_O_2_), a ubiquitous metabolite, to water (H_2_O) [1,21]. This reaction prevents lipid peroxidation, maintains membrane integrity, and preserves enzymatic function [22,23]. Second are the thioredoxin reductases (TrxR), which play a vital role in maintaining cellular redox homeostasis [24]. They catalyze the reduction in oxidized thioredoxin (Trx) utilizing NADPH, regenerating reduced thioredoxin (Trx). Trx is then able to reduce sulfide bonds in an array of cellular proteins [25].

Because they are highly reactive, many selenoproteins are localized to the ER and catalyze protein folding and other redox-dependent ER functions [21]. The ER is a more pro-oxidant environment than the cytosol, with a reduced to oxidized glutathione ratio (GSH/GSSG) ranging from 3:1 to 1:1 compared to cytosolic GSH/GSSG of 30:1 to 100:1 [26]. This allows for disulfide bond formation necessary for protein folding, which in many cases is facilitated by selenoproteins. For example, selenoprotein F (formerly selenoprotein 15) is part of the unfolded protein response and is induced in the setting of ER stress to ensure disulfide bond formation in prematurely released glycoproteins [27,28]. In addition to protein folding functions, selenoproteins regulate other aspects of ER function. Selenoprotein N (SelenoN) regulates the myocyte response to oxidative stress via control of ryanodine receptor calcium permeability [29,30]. Mutations in SelenoN cause congenital myopathies [24,31].

**Table 1:** Summary of selenoproteins and their functions.

Category	Selenoproteins
Antioxidants/Redox homeostasis	**Glutathione Peroxidases (GPx)** GPx1: Most abundant GPx, catalyzes reduction in H_2_O_2_ and low molecular weight hydroperoxides to H_2_O through glutathione oxidation [32,33]. GPx2: Localized to gastrointestinal epithelium and liver in humans [33,34]. GPx3: “Extracellular” GPx, secreted into plasma and other extracellular fluids, also binds basement membrane of epithelia in kidney, epididymis, bronchi, type 2 pneumocytes, and gastrointestinal tract [33,35]. GPx4: The only monomeric GPx and can be localized to cytosol, mitochondria, and nucleus. Can reduce complex lipid hydroperoxides using either glutathione or other protein thiols as substrate. It is the only GPx whose genetic deletion is lethal [33,36]. GPx6: Selenoprotein in humans does not contain Sec in mice. Unknown function [32,33]. **Thioredoxin reductases (TrxR)** TrxR1: Cytoplasmic, NADPH-dependent, and maintains a pool of reduced thioredoxin, contributing to reducing environment required for diverse protein functions. If absent or inhibited, glutaredoxin (Grx) will compensate and maintain reduced thioredoxin pool [37,38,39]. TrxR2: Contains mitochondrial target sequence, but alternative splicing can result in cytosolic forms [37,38]. TrxR3: Testis-specific, can reduce both thioredoxin and glutathione [37,38]. **Other Oxidoreductases** Selenoprotein O: Largest mammalian selenoprotein, localized to mitochondria, contains kinase-like domain with unknown target [40,41]. Selenoprotein W: Function unknown. Highest expression in muscle, heart, and brain [42,43].
Endoplasmic reticulum-associated	**Endoplasmic Reticulum-associated Degradation Pathway (ERAD)** Selenoprotein K: Involved in endoplasmic reticulum (ER) stress response, extracting misfolded proteins contributing to proteasomal degradation [44]. Selenoprotein F: Formerly named 15 kDa selenoprotein or Sep15, forms complex with UDP-glucose:glycoprotein glucosyltransferase (UGGT) to reglycosylate misfolded proteins [28,45]. Selenoprotein S: Facilitates translocation of misfolded proteins in ER lumen to cytosol for proteasomal degradation [46,47]. **Calcium Homeostasis** Selenoprotein M: Function unknown. Localized to perinuclear ER and shares significant sequence homology with selenoprotein F, but does not associate with UGGT. May regulate calcium release [48,49]. Selenoprotein N: ER-calcium sensor, enhances sarcoplasmic-endoplasmic reticulum calcium ATPase (SERCA) activity in low ER luminal calcium state [30]. Selenoprotein T: Subunit of oligosaccharyl transferase complex, N-glycosylating intracellular calcium channels to regulate activity as well as corticotropins for secretion. Gene knockout leads to embryonic lethality [50,51].
Selenoprotein synthesis and transport	**Selenoprotein synthesis** Selenophosphate synthetase 2: Catalyzes production of monoselenophosphate from selenide and ATP, a required step in selenocysteine synthesis and generation of tRNA^sec^ [52,53,54]. **Selenoprotein transport** Selenoprotein P: Secreted protein containing up to 10 Sec residues and the only selenoprotein containing more than one Sec. It is the source of Se distributed to target tissues [55].
Thyroid function	**Iodothyronine deiodinases** Three enzymes (D1–D3) that remove iodine to activate (T4 to T3) or inactivate (T4 to reverse T3, or T3 to T2) thyroid hormone [56].

All identified human selenoproteins contain a single Sec residue with the exception of selenoprotein P (SelenoP) [1,57]. Unique among selenoproteins, SelenoP contains a catalytic, thioredoxin-like *N*-terminal domain and up to nine Sec residues in its *C*-terminal domain. SelenoP transports selenium to tissues by binding various tissue-specific lipoprotein receptors, including apolipoprotein E receptor 2 (apoER2) [55]. The *C*-terminal domain undergoes proteolytic cleavage and endocytosis, serving as a store of Sec until cleavage by Sec lyase and de novo selenoprotein synthesis in the target tissue [1,58]. The *N*-terminal, Trx-like Sec is catalytically active and detoxifies lipid hydroperoxides [59].

The final category of selenoproteins, iodothyronine deiodinases, catalyze the conversion of thyroxine (T4) to its active form, triiodothyronine (T3), which is crucial for growth and development [24]. While the role of Se deficiency in thyroid disease is controversial, there is some evidence for selenium supplementation efficacy in autoimmune thyroiditis, though this may be more related to the role of selenoproteins in immune function and effects on anti-thyroid peroxidase antibodies than a direct effect on thyroid hormone synthesis [60].

Selenoproteins require complex, energy-intensive synthetic pathways and unique translational machinery. The high reductive potential of selenocysteine contributes to diverse biological functions of selenoproteins in antioxidant defenses, protein synthesis and quality control, intracellular calcium regulation, and thyroid hormone regulation. Many of these functions are critical to the health of the preterm infant. To understand how Se may exert these biological functions, we discuss the uptake/transfer, metabolism, and distribution of selenium in the fetus and newborn.

## 2. Tracing the Path of Selenium, from Soil to Fetus

Dietary Se intake largely depends on the Se content of the soil in which the source food is grown, which varies geographically [61]. Se can enter the body in both inorganic (selenite, selenate, selenide) and organic (selenomethionine, methylselenocysteine, selenocystamine) forms [61]. Se deficiency in North America is extremely rare among the general population. Preterm infants, however, are susceptible to Se deficiency due to the lack of Se accrual in the third trimester of pregnancy and dependence on PN postnatally [3,62,63]. Fetal Se accrual is largely based on Se content in maternal diet, which itself is dependent on the Se content of the soil in which the mother’s food is grown [63,64]. While Se-deficient soils exist in North America, globalization and the complexity of food sourcing prevent epidemics of Se deficiency in healthy adults on a large scale in the industrialized world [65,66]. In contrast, regions of the world with low soil Se content, combined with limited food imports from Se-rich regions, have seen periodic epidemics of diseases related to Se deficiency. Keshan disease, a cardiomyopathy arising from coxsackievirus infection in Se-deficient individuals, and Kashin-Beck disease, an osteochondropathy leading to necrosis of growth plates in Se-deficient children, are most prevalent in Tibet and along a band from the northeast to southwest borders of China with extremely low soil Se content [67,68]. Targeted Se supplementation in these regions has drastically decreased the prevalence of these diseases in recent decades [69,70].

Selenate (SeO_4_^2−^) and selenite (SeO_3_^2−^), the most common inorganic forms, are abundant in well-aerated soils and water [62,71]. These are absorbed by plants and converted into organic forms, which are the predominant source of Se in humans [62]. The most common dietary form of Se is selenomethionine (SeMet), which is found in cereals, legumes, some nuts, and animal products [62]. SeMet is structurally similar to methionine, with Se in place of sulfur (Figure 1), and when present in sufficient quantities, can nonspecifically replace methionine in proteins to serve as a Se storage pool, at a ratio ranging from 1:2800 to 1:8000 SeMet:methionine, depending on the amount of SeMet intake [62,72]. Sec is also present in the diet, though it exists almost exclusively within intact selenoproteins like the Gpx family, as free Sec is highly reactive and unstable at physiologic pH due to its aforementioned low pK_a_ [73].

Se metabolism occurs in four phases: intestinal absorption, hepatic uptake and selenocysteine synthesis, post-hepatic distribution for selenoprotein synthesis in target tissues, and excretion. Initial data on Se absorption relied on radioisotope tracing in either animals or adult participants, while more recent advances in sample purification and mass spectrometry have enhanced precision and enabled safe study of samples from blood or other body fluids [74,75,76,77,78,79,80]. The mechanism of intestinal Se absorption depends on the form of Se ingested. SeMet and SeO_4_^2−^ have bioavailability greater than 90% and use the same transport mechanisms as their sulfur-containing analogs Met and sulfate (SO_4_^2−^). These are rapidly absorbed into enterocytes by ATP-dependent active transport and into the bloodstream through passive basolateral transporters [74,75,76,79,81]. SeMet appears to be absorbed by the same transporter as Met, whose basolateral transporters belong to the L-type amino acid transporter (LAT) family [74,77,81]. Notably, LAT-1 transporter expression changes during normal gestation, moving from the cytosol to the cell surface during late gestation [82]. This raises the possibility that enteral SeMet absorption in preterm infants is less efficient than that of term infants or adults and may explain the persistence of low selenium in enterally fed and supplemented preterm infants [63].

Selenite (SeO_3_^2−^) is approximately 50% bioavailable when ingested in physiologic amounts, as it undergoes passive transport and is reduced to selenide (Se^2−^) in enterocytes, with minimal serum selenite present in the absence of supplementation [83]. Sec has not been detected in the bloodstream of humans, and Sec from digested selenoproteins likely undergoes reduction in enterocytes prior to absorption [75,83,84]. Humans lack selenomethyltransferases (SMTs), which convert Sec to methylselenocysteine (MSeC), a stable selenium form with high bioavailability in humans. Certain plants have SMTs, and MSeC can constitute up to 80% of plant Se (broccoli, radish, brussels sprouts, cabbage, garlic, onion, and leek). These compounds are absorbed and reduced in the liver to enter the Se pool in adults, but are present in minimal amounts in breast milk and are not added to PN [5].

**Figure 2:**
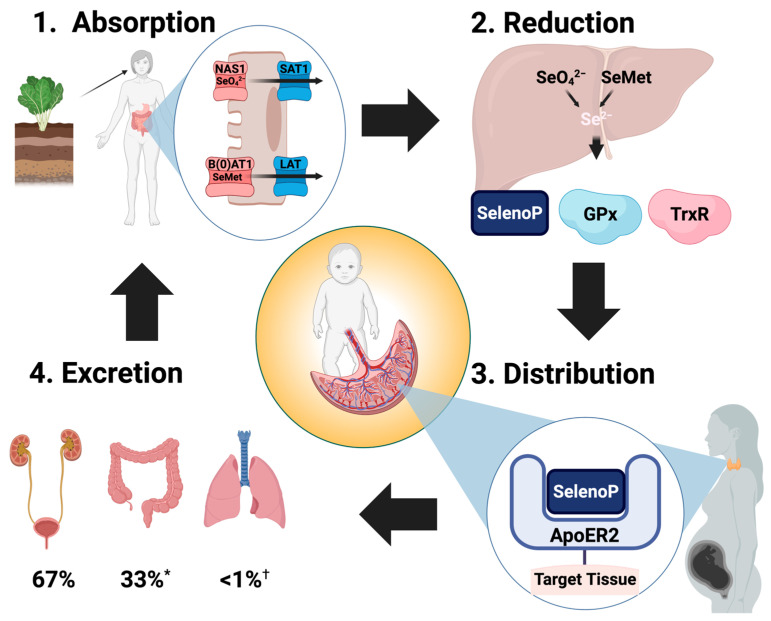
The nutritional cycle of Se begins with the ingestion of foods containing SeMet or through supplementation with selenate or selenite. Absorption occurs in the small intestine via apical transporters (red) and basolateral transporters (blue) that also function in sulfate and methionine absorption. In the liver, organic and inorganic sources of Se are reduced to selenide (Se^2−^) prior to de novo selenoprotein synthesis and distribution of Se by SelenoP. Target tissues take up SelenoP through apolipoprotein receptor-mediated endocytosis. In pregnant patients, target tissue Se uptake includes placental uptake of SelenoP by ApoE receptor 2 (ApoER2) for fetal transfer. Most Se is eliminated as selenosugars in the urine. NAS1—Na^+^-sulfate cotransporter; SAT1—sulfate anion transporter; B(0)AT1—Na^+^-dependent neutral amino acid transporter; LAT—L-type amino acid transporter. Created in BioRender. Mathias, M. (2026) https://BioRender.com/lvt7aua. * Because selenite has inefficient absorption with only 50% bioavailability, studies including enteral selenite supplementation find a significantly higher proportion of Se eliminated in the feces. ^†^ In the setting of toxic Se exposure, Se is eliminated through expiration as dimethylselenide, a volatile compound with a garlic-like odor.

Following absorption, SeO_4_^2−^ and SeMet enter circulation largely unchanged prior to uptake in the liver [1,61]. Within the liver, both inorganic and organic forms of Se undergo multi-step reduction to generate selenide, the most reduced form of Se (Figure 2). Post-hepatic Se is transported predominantly as SelenoP, which accounts for 40–60% of plasma Se. The remaining Se fraction is either albumin-bound, nonspecifically incorporated into proteins as SeMet, present as Gpx, or present in trace amounts as a variety of metabolites [24,83,85,86,87,88]. Additional mechanisms exist for Se transport and delivery, as Se can bind to other plasma proteins, including low-density lipoproteins (LDL), very low-density lipoproteins (VLDL), and alpha-globulin [73].

Circulating maternal SelenoP can be taken up by the placenta through receptor-mediated endocytosis, in a process involving apolipoprotein E receptor-2 and other low-density lipoprotein receptor proteins (LRP) like megalin (LRP2) [2]. Megalin is present in the visceral yolk sac; mouse studies indicate less importance than ApoER2 for fetal Se levels [2]. Mouse studies have also demonstrated that in the early- to mid-gestation phase, the visceral yolk sac is key to fetal Se supply and primarily mediated through maternal SelenoP and GPx3 uptake by epithelial cells through pinocytosis [2]. Once Se is taken up by the placenta, maternal SelenoP is catabolized and released to the fetal circulation [2]. Se is supplied for selenoprotein synthesis either through selenocysteine lyase (Sec lyase)-dependent pathways or Sec lyase-independent pathways, where SelenoP may deliver Se without undergoing degradation [85]. Maternal Se levels are directly correlated with gestational age, birth weight, and neonatal Se levels, while low maternal Se is independently associated with increased risk of preterm birth [89].

Data on Se excretion are challenging to interpret, as the form and amount of Se intake influence the route of excretion [90]. Studies on Se elimination that used enteral selenite supplementation found significantly more excretion in the feces, likely due to the lower bioavailability relative to SeMet and SeO_4_^2−^ [84,91]. In contrast, studies of adult men and women with organic Se supplementation found that half to two-thirds of Se was excreted in the urine (Figure 2) [75,92]. As many as 16 different Se-containing urinary metabolites, predominantly selenosugars, have been identified [93,94,95,96]. These are thought to be generated as byproducts of the multistep reduction to selenide on the inner mitochondrial membrane [90,97]. There are limited data on urinary excretion of Se in infants, though a small study of enterally fed Japanese infants found significantly higher urine Se in preterm infants compared to term infants at a week of age, potentially contributing to the significantly higher risk of selenium deficiency in preterm infants [98].

The fetus and preterm infant pose unique nutritional challenges due to their high energy consumption, exposure to oxidative stress, and developmentally immature intestinal absorption and feeding abilities. Supplementation of selenium to overcome these challenges requires careful consideration of the dose, the form of Se, and how to define adequacy and deficiency in this population.

## 3. Selenium in Enteral and Parenteral Nutrition of Preterm Infants

Se requirements increase both during pregnancy and after birth during lactation, with the recommended dietary allowance (RDA) of 60 µg/day for pregnant women and 70 µg/day when breastfeeding. In contrast, the standard RDA of Se is 55 μg/day [62,99,100]. Reduced maternal Se levels have been associated with pre-eclampsia, in utero growth restriction, fetal malformations, impaired neurodevelopment, and increased infection risk [100]. Even for full-term infants, there are insufficient data to determine a recommended dietary allowance of Se under one year of age [62,99]. Instead, a flat “estimated average requirement” of 15 μg is recommended, reflecting a lower level of evidence than that of adult RDAs. This amount is based on estimated intake and average Se content in human milk for exclusively breastfed infants, largely from studies conducted in the 1980s and 1990s [99,101,102,103,104,105,106,107]. Furthermore, even these estimates are weight-adjusted from adult requirements, which may overestimate or underestimate the distinct metabolic needs and oxidative stressors of term and preterm infants [99].

This scarcity of data to support recommendations for the RDA for Se is compounded by the complexity of determining Se adequacy or deficiency. In adults, the most abundant stores of Se are in skeletal muscle, followed by the bone, liver, and kidneys, with estimates of blood Se as a proportion of whole-body Se ranging from 1% to 10% [108,109]. Term and preterm newborn body composition is dramatically different from that of adults, particularly the lack of muscle and bone tissue, with rapid changes over the first weeks and months of life [110]. Solid organ Se distribution in a small study of infants found significant differences in Se distribution, with much higher levels of Se in the kidneys as a proportion of total body Se [111]. In states of deficiency, the distribution of Se throughout the body changes to prioritize critical functions, referred to as the “selenoprotein hierarchy” [1]. Mice fed Se-deficient diets depleted their liver Se stores and increased brain Se content as a proportion of total body Se [57]. Se distribution between specific selenoproteins is also not stable. When Se is repleted in Se-deficient adults, GPx expression and activity plateaus at lower serum Se levels than does SelenoP [112].

The three most abundant forms of Se in the blood are SelenoP (50%), Gpx (40%), and albumin-bound Se (10%) [113]. Given their relative sensitivity to Se supplementation, red blood cell Gpx activity and serum SelenoP levels have been proposed as biomarkers of Se status [57,112]. Preterm infants have lower serum albumin content and Gpx levels when compared to term infants, and Se reference ranges would first need to be established in this population prior to clinical use of these biomarkers in this population [114,115,116,117]. Red blood cell Gpx activity has not been shown to reliably predict Se status in preterm infants [116]. Lastly, approximately two-thirds of preterm infants born under 32 weeks’ gestation receive blood transfusions during their hospital stay, confounding infant blood Se levels with the introduction of adult red blood cells [118]. Other forms of Se monitoring, including urine, fecal, and hair or nail Se content, have been proposed. Urine, hair, and nail Se content varies based on the form of Se intake [119,120]. While meconium Se content may reflect the accrual of Se during late gestation, it is impractical to depend on stool Se in parenterally fed infants, as this measurement is unlikely to reflect true Se status.

Approximately two-thirds of the Se contained in breast milk is in the form of the organic selenoproteins Gpx3 and SelenoP [5,121,122]. As outlined above, these SeC- and SeMet-containing proteins undergo reduction to elemental selenide via the transsulfuration pathway prior to incorporation into de novo selenoproteins in the liver (Figure 1) [1,21,61]. Curiously, breast milk also contains significant amounts of the oxidized selenometabolite, selenocystamine, which is not present in blood but comprises up to 25% of breast milk Se [5,122,123,124]. In vitro, selenocystamine oxidizes glutathione and generates superoxide radical, though only when present at concentrations 1000-fold higher than those found in breast milk and blood [125,126]. Selenocystamine has also been shown to reduce ferroptosis in a rodent model of ischemia–reperfusion [127]. In contrast to the more abundant organic forms of Se, SeC, and SeMet, selenocystamine requires reduction by glutathione in order to be incorporated into the Se pool [1]. It is not known if the superoxide-generating or anti-ferroptotic properties of selenocystamine play a biological role in breast milk; however, this selenocompound, while relatively abundant in human milk, is not present in infant formulas or commercially available trace element formulations. Unlike the organic Se forms in breast milk, the Se in commercially available infant formula and PN is in the form of selenous acid (H_2_SeO_3_), sodium selenite (Na_2_SeO_3_), or sodium selenate (Na_2_SeO_4_) [128,129,130,131]. These forms rely on reduction by glutathione (GSH) prior to incorporation into de novo selenoprotein synthesis.

**Figure 3:**
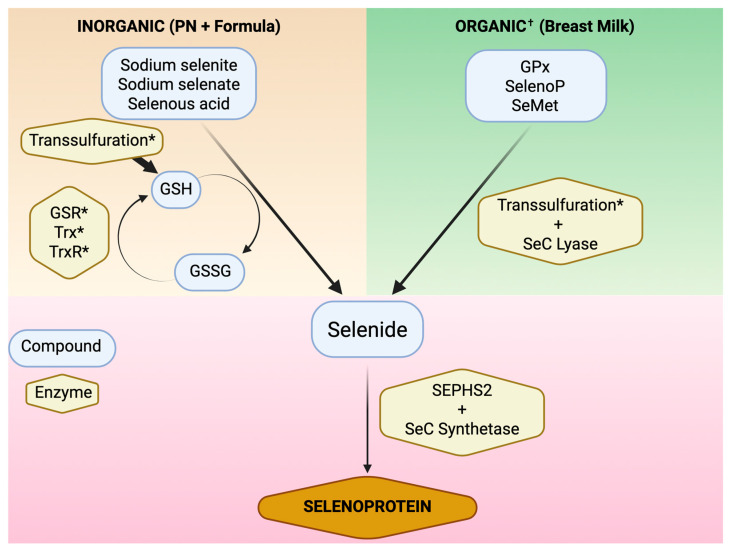
Schematic of different pathways of Se metabolism and incorporation into de novo selenoproteins. All Se is reduced to selenide prior to incorporation into selenocysteine and selenoproteins. Inorganic sources of Se depend on glutathione for reduction to selenide.; Trx—thioredoxin; TrxR—thioredoxin reductase; GPx—glutathione peroxidase; SelenoP—selenoprotein P; SeMet—selenomethionine; SeC—selenocysteine; SEPHS2—selenophosphate synthetase 2. Created in BioRender. Mathias, M. (2026) https://BioRender.com/qehrkfg. * Deficient in preterm infants. ^†^ Oxidized organic selenometabolites like selenocystamine undergo GSH-dependent reduction prior to transsulfuration. GSH—glutathione; GSSG—glutathione disulfide; GSR—glutathione reductase.

Because inorganic Se undergoes GSH-dependent metabolism, it may not be the optimal form of Se supplementation in preterm infants, as GSH synthesis and metabolism are impaired in this population (Figure 3) [132]. In a small cohort of maternal-infant dyads, cord blood cysteine and GSH levels were significantly lower in preterm infants compared to term infants, while erythrocyte GSH synthetic capacity was not [133]. Because GSH redox homeostasis and selenoprotein synthesis are interdependent, dietary or parenteral supplementation to “optimize” GSH is an attractive solution to ensuring adequate selenoprotein bioactivity. Adequate provision of the GSH peptide precursors glycine, glutamate, and cysteine is necessary for GSH synthesis in early life, and supplementation above recommended amounts may increase total glutathione synthesis in preterm infants [134,135,136]. Among the three peptide precursors, cysteine is the rate-limiting substrate for GSH synthesis [137,138]. However, a randomized, controlled trial of high-dose cysteine supplementation in preterm infants did not result in increased glutathione [139]. In a Cochrane review, supplementation of cysteine, cystine, and N-acetylcysteine did not result in significant increases in GSH or in any of the clinical outcomes measured [140]. Direct supplementation of GSH in parenteral nutrition is feasible and has been shown to mitigate the damaging effects of photo-oxidized ascorbic acid in a guinea pig model [141,142]. While increasing GSH availability may mitigate the damaging effects of reactive oxygen intermediates in preterm infants, effective and reliable strategies have yet to be identified.

While Se toxicity, or selenosis, has been described since the 13th century, Se was not identified as an essential mineral in mammals until 1957 [143]. PN was introduced in the United States in the 1960s, but it was not until 1979 that the American Medical Association recommended routine addition of the trace elements zinc, copper, manganese, and chromium [144,145,146,147]. That same year, a case series of post-operative patients on 10 or more days of PN described symptoms of muscle pain, weakness, and difficulty walking that improved with Se supplementation [148]. In 1983, a 6-year-old child on more than 1 year of PN developed muscle pains, elevated creatine kinase, elevated transaminases, and fingernail pigmentation changes and was found to be Se deficient [149]. He was supplemented with selenous acid, and laboratory values and fingernail changes improved. The first universal recommendation to add Se to PN in the United States occurred in 1984 [147,150]. Low serum Se has been associated with bronchopulmonary dysplasia, a respiratory disease that disproportionately affects infants born extremely preterm [151,152,153]. Perinatal dietary Se deficiency has been shown to disrupt lung and pulmonary vascular development and exacerbate hyperoxic lung injury [154,155]. There may be additional benefits to Se supplementation in specific clinical scenarios. Selenoprotein-dependent antioxidant systems can be variably induced and suppressed in sepsis [109,156]. A study of adults with severe sepsis found improved mortality with administration of high doses of sodium selenite (1 g/day for 10 days) [157]. A Cochrane meta-analysis from 2003 of three trials of Se supplementation in preterm infants found a relative risk reduction of 27% for one or more episodes of sepsis and a number needed to treat of 10 to prevent one or more episodes of sepsis [158]. Of note, two of the three trials included in this meta-analysis were from regions with low Se among the general population. In 2012, the American Society for Parenteral and Enteral Nutrition (A.S.P.E.N.) and the American Society of Clinical Nutrition recommended a dose of 2 μg/kg/day in term neonates and 1.5–4.5 μg/kg/day in preterm infants [147].

The only commercially available, FDA-approved multiple trace element formulation for preterm infants in the United States contains 2 μg/kg/day when administered per the manufacturers guidelines [128]. It is unclear if this dose prevents postnatal Se deficiency. A study of infants on 30 days or more of PN receiving 2–3 μg/kg/day parenteral Se found that those born ELBW and VLBW were nearly all Se deficient at the time of first measurement and were 16–17 times as likely to develop Se deficiency [4]. This publication may not be generalizable to all preterm infants, as most do not receive 30 days or more of parenteral nutrition. In a 2008 study on enteral Se supplementation, VLBW infants were deficient at birth and became more deficient in the first weeks of life [63].

In contrast to the ASPEN recommendations, European/Chinese guidelines recommend 7 μg/kg/day in parenteral nutrition for preterm infants (Table 2) [159]. This was based on the largest randomized controlled trial (RCT) of Se supplementation in preterm infants, which found that 7 μg/kg/day IV supplementation in preterm infants was required to prevent biochemical Se deficiency, defined as plasma Se < 45 μg/L [160]. While there were no differences in primary outcomes of death or oxygen dependence at 28 days, there was a statistically significant relative risk reduction of 25% in late-onset sepsis (*p* = 0.038) [160]. Of note, this study used sodium selenate (Na_2_SeO_4_), so any Se not taken up by the liver would be rapidly excreted in urine via sulfate transporters [84,87]. A Cochrane review and meta-analysis combined this study with two smaller trials and found a pooled relative risk of one or more episodes of sepsis of 0.73 (CI 0.59 to 0.93) without significant heterogeneity [158,161,162]. As the authors note, two of the three included studies were performed in regions with low soil Se content (New Zealand and Australia) and might not be generalizable to a European or North American population. We found one subsequent RCT by Aggarwal et al. in 2016, which reports a significant reduction in culture-proven late-onset sepsis among 90 infants born at 1000–1499 g randomized to receive either 10 μg/day enteral supplementation or placebo (Table 2) [163]. However, this study does not report the form of Se supplemented, and there is a notable, though not statistically significant, difference in the duration of ruptured membranes of 9.06 ± 24.17 h in Se-treated and 23.26 ± 57.51 h in placebo.

There is evidence that most extremely preterm infants are born with biochemical Se deficiency (plasma Se < 45), with the caveat that ‘normal’ fetal plasma Se has not been defined [171]. Because fetal Se accrual occurs via endothelial transporters (SeMet) and endothelial lipoprotein receptor endocytosis of maternal SelenoP, Se accrual is likely to be impaired in fetal growth restriction, preterm birth, and/or hypertensive diseases of pregnancy associated with placental vascular maldevelopment [172]. This impaired Se accrual is compounded by decreased expression and activity of both selenoprotein and other antioxidant enzymes, and concurrent exposures to reactive oxygen intermediates in infants born preterm [173].

The existing data are conflicting in how to define deficiency or adequacy of Se in preterm newborns. When Se was initially identified as an essential trace element, studies noted a linear relationship between supplementation and GPx activity [174,175]. In the largest randomized controlled trial including preterm infants, plasma GPx expression in supplemented infants was higher than in unsupplemented [160]. However, other authors have found no correlation between plasma or erythrocyte Se and GPx activity in preterm infants [176]. These same authors noted in a subsequent study that in term infants, enteral Se supplementation was correlated with erythrocyte GPx activity but not plasma GPx activity [177].

**Figure 4:**
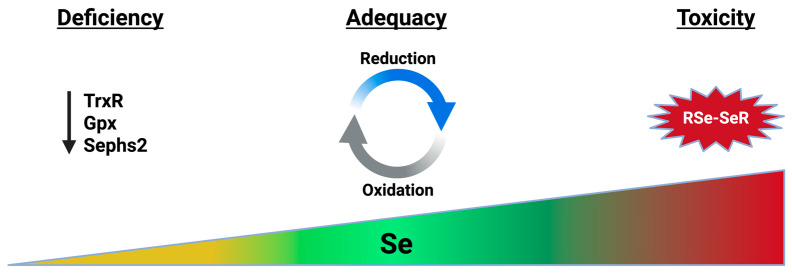
Adequate selenium is necessary for the synthesis and activity of many redox-active proteins. Se deficiency decreases the expression and activity of selenoproteins with diverse biological roles. Excess Se can generate unstable toxic compounds, including diselenides, leading to nonspecific oxidative damage to cells. Created in BioRender. Mathias, M. (2026) https://BioRender.com/qdsgmbc.

At the time of this writing, only testing for plasma or serum Se levels is commercially available, and we are not aware of any randomized controlled trials of individualized targeted Se supplementation in infants to achieve a threshold plasma level or GPx activity level. In a Se-deficient cohort of Chinese adults (defined by daily intake <14 μg/day in a region with endemic deficiency), selenoprotein P was the most sensitive biomarker to changes in Se supplementation [112]. Plasma GPx activity reaches a maximum and plateaus after a small amount of selenomethionine supplementation in a deficient population (35 μg/day, below the USDA RDA of 55 μg/day), while selenoprotein P reaches its maximum and plateaus at 93 μg/day [99,112]. This finding is consistent with the theory of selenoprotein hierarchy, where Se is preferentially distributed to selenoproteins and tissues with critical survival functions in states of deficiency [1]. In mice fed a Se-free diet for 4 weeks, total body Se decreased by 88%, but the decrease was predominantly in the liver and kidneys, with relatively preserved Se content in the brain, testes, and, to a lesser extent, muscle [55]. A uniform increase in Se supplementation for preterm infants, as is recommended by European and Chinese nutritional societies, may benefit preterm infants on a population scale; however, it is important to consider the form of Se and clinical context. Monitoring levels of glutathione and its substrates, as well as potential methylation products, may provide a more comprehensive evaluation of the effects of Se supplementation on this vulnerable population (Figure 4).

## 4. Conclusions

Selenoproteins serve critical functions in term and preterm newborn physiology owing to their diverse functions in antioxidant defenses, growth, and metabolism. Limited studies on term and preterm infants demonstrate a higher likelihood of Se deficiency in this population. Nonetheless, the only FDA-approved trace element formulation for neonatal PN in the United States might not adequately supplement Se levels in select populations. Measurement of Se stores remains a practical challenge, presenting a barrier to assessing the adequacy of current supplementation strategies. While blood (both plasma and erythrocyte-specific) GPx activity may correlate with Se supplementation in deficient preterm infants, GPx activity is likely to plateau at levels below those needed for adequate target tissue selenoprotein function. Total plasma or serum Se is likely dependent on the form of Se supplementation. Plasma Se continues to rise with SeMet supplementation due to nonspecific incorporation at methionine sites and may not correlate with target tissue selenoprotein activity. SelenoP is a promising biomarker for selenium deficiency and adequacy, as it is saturable and biologically plausible to correlate with target tissue selenoprotein levels. However, tissue-specific expression of apolipoprotein receptors in different physiologic states may affect tissue distribution of Se at the same concentration of SelenoP. A “gold standard” would require testing multiple forms of Se supplementation and multiple biomarkers or surrogates of Se and correlating these with clinical outcomes, applying multiple regressions to delineate which forms and biomarkers were best fit to clinical outcomes, and then prospectively comparing a targeted and untargeted approach to Se supplementation using these models. Absent such a resource-intensive investigation, the Darlow et al. 2000 [160] study is the most robust data we have showing a clinical benefit of uniform Se supplementation in preterm infants and those requiring parenteral nutrition, and serves as the primary source of consensus recommendations for universal Se supplementation in preterm infants.

Incorporation of current formulations of inorganic Se in PN requires antioxidant pathways that have been shown to be deficient in preterm infants. Reliable testing of neonatal Se stores that does not require large volumes of blood would permit further study and individualized recommendations for Se supplementation. Significant progress has been made in understanding the need for Se in enteral and PN since the 1984 AMA recommendation, and current guidelines are likely to prevent cases of severe Se deficiency described prior to its routine addition to PN. Future improvements to testing and clarity on the individual needs of Se and selenometabolites may serve to further optimize nutrition in the newborn.

## Figures and Tables

**Table 2:** Enteral and parenteral selenium supplementation guidelines for preterm infants.

Society	Recommendation	Sources and Notes
ESPGHAN/ESPEN/ESPR/CSPEN * Domellöf et al. 2018 [159]	Parenteral: 7 μg/kg/day	Darlow et al. 2000 [160] - Dose: Selenate 7 μg/kg/day, RCT of 534 infants < 1500 g at birth in New Zealand, where soil Se content is low. - Outcomes: Primary—No change in O_2_ dependency at 28 days. Secondary—Increased plasma Se and GPx activity in supplemented infants.
ESPGHAN Embleton et al. 2023 [164]	Enteral: 7–10 μg/kg/day	Tyrala et al. 1996 [165] - Dose: Selenate 3–5 μg/kg/day, RCT of 17 infants < 33 weeks and <2000 g at birth in Pennsylvania, formula only. - Outcomes: Primary—Increased plasma GPx activity, RBC and plasma Se content. Secondary—no difference in anthropometrics.
		Darlow et al. 2000 [160] - Dose: Selenite 5 μg/kg/day, estimated 7 μg/kg/day total with breast milk. - Outcomes: See above.
		Aggarwal et al. 2016 [163] - Dose: Se form not specified 10 μg/day, RCT of 90 infants 1000–1499 g and <32 weeks at birth in Rohtak, Haryana, India. - Outcomes: Primary—Decreased late onset sepsis in 6/45 placebo-treated infants and 0/45 Se-treated infants. Secondary—Increased serum Se level, decreased “probable” sepsis in treated group.
ASPEN ^†^ Vanek et al. 2012 [147]	Parenteral: 1.5–4.5 μg/kg/day Enteral: 1.3–4.5 μg/kg/day	No direct references to clinical trials including preterm neonates. Greene et al. 1988 [166] and Tsang et al. 2005 [167] are cited as sources of these recommendations and are a committee report from the American Society of Clinical Nutrition and a book chapter, respectively.
ASPEN Barbieri and Cober 2023 [168]	Parenteral: 7 μg/kg/day	Darlow and Austin 2003 [158], Cochrane review and meta-analysis which includes 3 studies [160,161,162]. A total of 583 total infants, significant reduction in one or more episodes of sepsis with Se supplementation.
		Huston et al. 1991 [162] - Dose: Selenous acid (selenite) 1.5 μg/kg/day, RCT of 20 infants < 1000 g at birth in Oregon. - Outcomes: Increased serum Se at time of initiation of oral feeds and increased white blood cell GPx activity at initiation of oral feeds and at full oral feeds.
		Daniels et al. 1996 [161] - Dose: Selenous acid (selenite) 3 μg/kg/day, RCT of 38 infants < 2000 g at birth in Australia. - Outcomes: Increase in plasma Se over 3 weeks of supplementation that was not sustained at 6 weeks of supplementation.
		Darlow et al. 2000 [160], see above.
	Enteral: 7–10 μg/kg/day RDA, supplementation beyond routine fortification not recommended.	Finch 2015 [169], a review citing Darlow et al. 2000 [160] (see above) and Masumoto et al. 2007 [170], a retrospective chart review of 6 infants with clinical Se deficiency.
		Aggarwal et al. 2016 [163], see above.

* The European Society for Paediatric Gastroenterology Hepatology and Nutrition/European Society for Clinical Nutrition and Metabolism/European Society for Paediatric Research/Chinese Society for Parenteral and Enteral Nutrition. ^†^ American Society for Parenteral and Enteral Nutrition. Vanek et al. 2012 is the most recent ASPEN position paper with Se recommendations for preterm infants [147]. Barbieri and Cober 2023 is an invited review in the ASPEN journal *Nutrition in Clinical Practice* [168].

## Data Availability

No new data were created or analyzed in this study. Data sharing is not applicable to this article.

## Abbreviations

The following abbreviations are used in this manuscript:

Se	Selenium
Sec	Selenocysteine
PN	Parenteral nutrition
tRNA^sec^	Selenocysteine transfer RNA
eEFSec	Selenocysteine transfer RNA elongation factor
SECIS	Selenocysteine insertion sequence
SBP2	Selenocysteine insertion sequence binding protein 2
ER	Endoplasmic reticulum
Gpx	Glutathione peroxidase
H_2_O_2_	Hydrogen peroxide
TrxR	Thioredoxin reductase
Trx	Thioredoxin
NADPH	Reduced nicotinamide adenine dinucleotide phosphate
GSH	Glutathione, reduced
GSSG	Glutathione, oxidized
UDP	Uridine diphosphate
SelenoP	Selenoprotein P
T3	Triiodothyronine
T4	Thyroxine
SeO_4_^2−^	Selenate
SeO_3_^2−^	Selenite
SeMet	Selenomethionine
LAT	L-type amino acid transporter
NAS1	Sodium-sulfate cotransporter
SAT1	Sulfate anion transporter
B(0)AT1	Sodium-dependent neutral amino acid transporter
SMT	Selenomethyltransferase
MSeC	Methylselenocysteine
LDL	Low-density lipoprotein
VLDL	Very low-density lipoprotein
LRP	Lipoprotein receptor protein
ApoER2	Apoplipoprotein E receptor 2
SEPHS2	Selenophosphate synthetase 2
RDA	Recommended daily allowance
ESPGHAN	The European Society for Paediatric Gastroenterology, Hepatology, and Nutrition
ESPEN	The European Society for Clinical Nutrition and Metabolism
ESPR	The European Society for Paediatric Research
CSPEN	The Chinese Society of Parenteral and Enteral Nutrition
ASPEN	The American Society for Parenteral and Enteral Nutrition
